# Intracranial Penetrating Taser Dart Injury

**DOI:** 10.7759/cureus.87024

**Published:** 2025-06-30

**Authors:** Christopher Riba, Emmelyn Samones, W. Seth Dukes

**Affiliations:** 1 Emergency Medicine, Loma Linda University Medical Center, Loma Linda, USA

**Keywords:** case report, conducted electrical weapon, penetrating intracranial injury, taser, trauma

## Abstract

A healthy 52-year-old male presents to the emergency department after being tased and found to have an intracranial penetrating taser dart injury to the left frontal bone. The patient displayed somnolence with disorientation but no focal neurologic deficits on initial evaluation. Radiograph and computed tomography imaging demonstrated taser penetration into the cranial vault without clear evidence of active hemorrhage. Management with neurosurgery was planned; however, the patient eloped before intervention, a decision that could have had severe consequences. The patient later reported that the barb was removed in a nonmedical setting and denied known complications.

Tasers can penetrate the skull and intracranial vault, posing risks of hemorrhage and infection. Although the patient reported no medical complications, emergency physicians remain vigilant for such sequelae during patient evaluation.

## Introduction

The taser is a conducted energy weapon and is a nonlethal alternative to firearms commonly used by law enforcement. It incapacitates targets by launching two barbed projectiles that deliver numerous electrical pulses that stimulate motor neurons, inducing pain and transient paralysis. While designed to be nonlethal, injury from taser use is not unheard of, and many post-tasered individuals are taken to the emergency department to be evaluated for related injuries [1,2]. Currently, routine laboratory tests, electrocardiograms, and imaging are not recommended, with more extensive testing being used based on case circumstances and clinical judgment [3]. In this report, we present a unique and unexpected case of a man brought to our facility after tasing and found to have an intracranial penetrating taser injury.

## Case presentation

A 52-year-old male without known significant past medical history arrived by ambulance due to concern for a head injury. Per emergency medical services (EMS), the patient was involved in a foot chase with police, during which the patient was tased and apprehended. The taser barb struck the patient in the forehead and back. During evaluation by police, the patient displayed waxing and waning consciousness and was oriented only to self. At that time, the patient endorsed only drinking alcohol. Still, per EMS, a bystander at the scene with the patient reported the patient had also recently smoked methamphetamine and marijuana. Despite these circumstances, the patient was cooperative and was subsequently brought to the emergency department for further evaluation.

**Figure 1:**
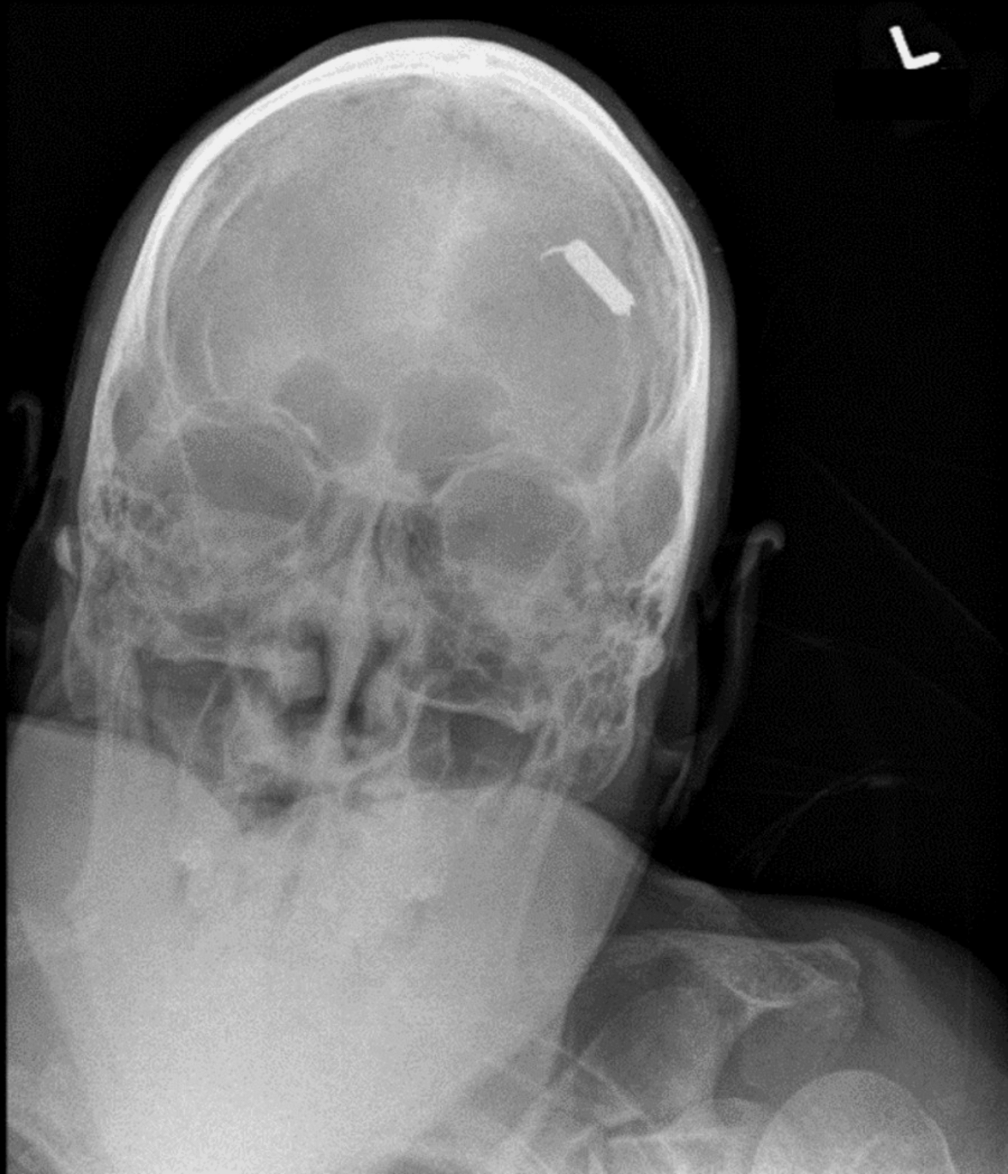
Radiograph of the skull displaying taser barb projecting over the left frontal bone

Upon intake, the patient’s vital signs were unremarkable; however, the physical examination revealed significantly reduced responsiveness, with orientation limited to self and year corresponding to a Glasgow Coma Scale of 13 (eye = 3, verbal = 4, motor = 6). No focal neurologic deficits were noted, and a single taser barb was identified embedded within the left side of the patient’s forehead. Removal attempts were halted appropriately due to resistance. A radiograph of the skull revealed a taser barb penetrating deep to the calvarium without other fractures noted; possible intracranial penetration was not excluded (Figure 1).

**Figure 2:**
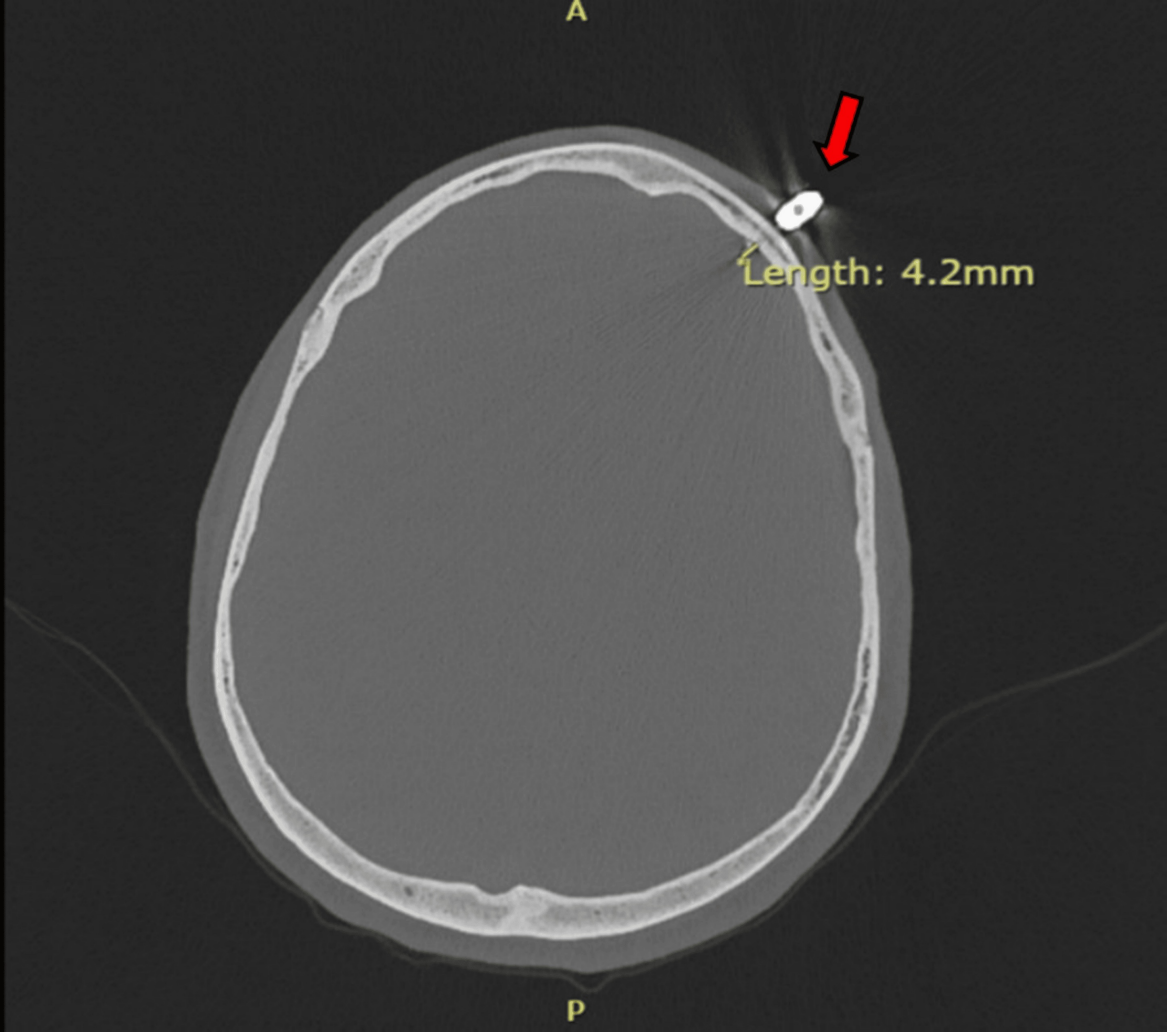
Computed tomography angiogram of the head with and without contrast displaying metallic foreign body embedded within the left frontal cranium (red arrow) and extending 4 millimeters intracranially, beyond the inner table

**Figure 3:**
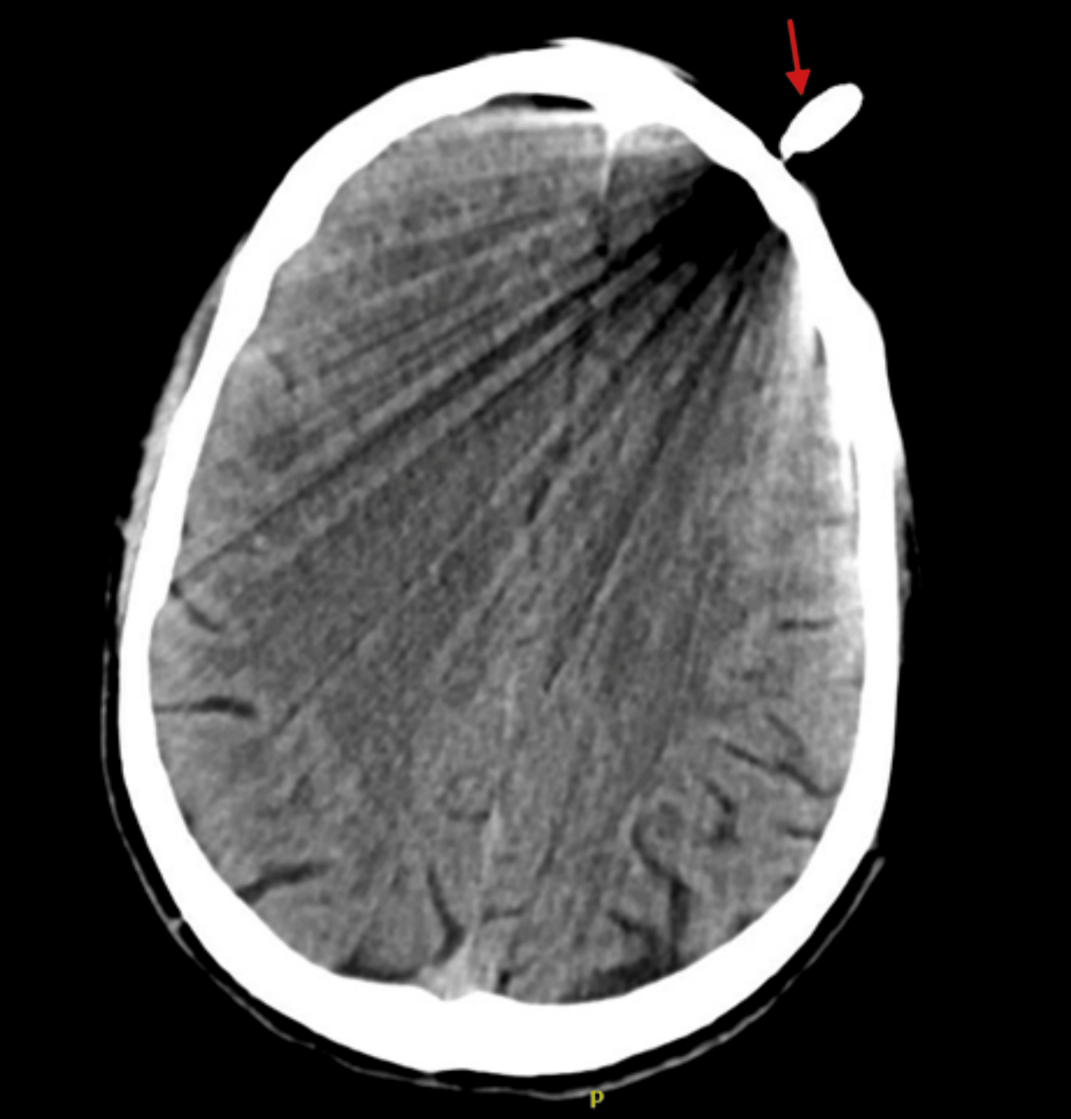
Computed tomography angiogram of the head with and without contrast with brain displaying metallic foreign body embedded within the left frontal cranium (red arrow) with significant beam artifacts

The patient, in critical condition, was subsequently trauma activated, and consults to trauma surgery and neurosurgery were placed. According to neurosurgery's recommendations, a computed tomography (CT) angiogram of the head, with and without contrast, was necessary for further characterization. Results of the imaging found that the taser bolt extended 4 millimeters intracranially, beyond the inner table of the cranial vault (Figures 2, 3).

There was no evidence of extra-axial contrast extravasation, suggesting that an acute hemorrhage was unlikely to have occurred. However, the beam artifact from the metallic dart limited the detection of subtle hemorrhage. Given these findings, neurosurgery recommended admission for surgical removal of the barb, with intravenous ceftriaxone administered for infection prophylaxis. The patient eloped from the emergency department before these interventions were performed, and all attempts to contact the patient at that time were unsuccessful.

One year after these events, the patient was successfully contacted and provided information about what occurred following his elopement from the emergency department. The patient reported that several hours after he left, he met with several people, all of whom attempted to remove the barb. Ultimately, one individual was able to rip the barb free using a pair of pliers but found that the tip of the barb appeared to be missing. The patient denied subsequent healthcare visits for fevers, headaches, altered mentation, neck stiffness, or other symptoms of infection or hemorrhage. He denied recurrent headaches since this incident and stated that the only remaining evidence of his injury is a small, dense subdermal "bead" that is palpable over his left forehead. The patient believes the “bead” is a small piece of metal that remains in his forehead.

## Discussion

This case underscores the rarity of a complication of taser use: skull penetration by a bolt with intrusion into the cranial cavity. It illustrates a scenario where no medical intervention was undertaken, yet the patient survived without reported complications. The taser device administers its incapacitating electrical current through two metal barbed projectiles, each nine millimeters in length. These are launched via travel distances, the travel of roughly 7-11 meters at a velocity of 250 feet per second [1,2]. The force with which these barbs are launched is designed to allow secure penetration of the clothing and epidermis, enabling continual metal contact while administering the electrical impulses. While not intended, the produced forces have been reported to break bones and occasionally injure deeper structures [4,5]. Research using human skulls has demonstrated that it is theoretically possible for taser barbs to fracture and even pierce the human cranium [6]. However, this case illustrates a rare real-world instance of this occurring.

It is imperative that patients who present after being tased are initially evaluated and resuscitated as needed using the standard advanced trauma life support and advanced cardiac life support. If there is any difficulty removing the barb, it is recommended that all attempts to remove the taser barb be halted until radiograph or CT imaging is obtained to evaluate for penetrance into other structures, such as bone [3,7-9]. Should this complication be identified, it is important to contact surgical specialists early, as retained metallic fragments in the cranium can result in traumatic brain injury, infection, and seizures [10,11]. This case report supports this practice and demonstrates that while taser darts are not intended to produce forces capable of serious injuries, they can produce sufficient energy to penetrate even the cranial vault. All emergency medicine physicians should be mindful of this possible complication while evaluating their patients.

## Conclusions

This case demonstrates that tasers can produce sufficient force to pierce through the skull and penetrate the vault. While the patient reported no medical complications, the risks of this injury include significant intracranial hemorrhage and infection. For this reason, emergency medicine physicians should be cognizant of this possible complication of taser use during evaluation and have a system-based response prepared that includes protocolized imaging and specialist consultation.
